# Changing the Inoculum Type From Preculture to Spore Suspension Markedly Alters the Production of Secondary Metabolites in Filamentous Microbial Coculture

**DOI:** 10.1007/s00284-024-04007-x

**Published:** 2024-12-07

**Authors:** Tomasz Boruta, Weronika Pawlikowska, Martyna Foryś, Grzegorz Englart, Anna Ścigaczewska

**Affiliations:** Department of Bioprocess Engineering, Faculty of Process and Environmental Engineering, Lodz University of Technology, ul. Wólczańska 213, 93-005 Łódź, Poland

## Abstract

The shake flask cocultures of *Aspergillus terreus* and *Streptomyces rimosus* were investigated with regard to the production of mevinolinic acid (lovastatin), oxytetracycline, and other secondary metabolites (SMs). The aim of the study was to determine the effect of inoculum type (spore suspension or preculture) on the levels of SMs in the fermentation broth. Altogether, 17 SMs were detected, including 4 products with confirmed identities, 10 putatively annotated metabolites, and 3 unknown molecules. As observed over the course of qualitative and quantitative analyses, the selection of inoculum type markedly influenced the SM-related outcomes of cocultures. Depending on the coculture initiation procedure, replacing the preculture with spore inoculum positively affected the biosynthesis of oxytetracycline, butyrolactone I, (+)-geodin, as well as the molecules putatively identified as rimocidin, CE-108, and (+)-erdin. It was concluded that the comparative analyses of SM production in filamentous microbial cocultures and monocultures are dependent on the type of inoculum and thus the diversification of inocula is highly recommended in such studies. Furthermore, it was demonstrated that designing a coculture experiment that involves only a single type of inoculum may lead to the underestimation of biosynthetic repertoires of filamentous microorganisms.

**Supplementary Information:**

The online version contains supplementary material available at 10.1007/s00284-024-04007-x.

## Introduction

Microbial secondary metabolites (SMs) constitute a large and still underexplored group of structurally diverse molecules of potential pharmaceutical interest. While not being directly involved in growth and energy metabolism, SMs allow their producers to thrive in environmental niches and engage in various interactions with other organisms. Their production, however, requires specific signals associated with medium composition, pH, temperature, aeration, developmental stage, morphological characteristics, presence of elicitors, etc., which collectively trigger the formation of SMs [1–3]. Providing these stimuli by employing conventional laboratory cultivation techniques is not always possible. Numerous strategies have been devised to awake the formation of SMs, e.g., genetic engineering of microbial strains or the modification of medium composition and bioprocess conditions [4–7]. Performing microbial cocultures is a convenient way to induce the production of previously unknown SMs as it allows to mimic the interspecies interactions that occur in natural habitats. What is more, this experimental approach does not require the molecular-level engineering or extensive biological knowledge to trigger the biosynthesis of novel SMs [8–10].

One of the key factors affecting the outcome of filamentous microbial cocultures (i.e., the ones involving fungi and/or actinomycetes) is the inoculation approach. Several aspects of coculture initiation need to be considered, including the choice of inoculum type (preculture or spores), its volume, and the time of inoculation (simultaneous or with the delayed inoculation of one of the strains). Many of the previously described filamentous cocultures were initiated using the precultures rather than spore suspensions [11–15]. Nevertheless, the reports on the use of spore suspensions for coculture inoculation can also be found in literature [16, 17]. Typically, the reasons for choosing either preculture or spore suspension for coculture initiation are not addressed in the studies dealing with the induction of SM biosynthesis. Even though this issue may at first seem relatively irrelevant in the broader context of discovering novel molecules and drug leads, it is known that the type of inoculum determines the results of SM-oriented fermentation processes and therefore this issue should not be overlooked [18, 19]. In principle, one cannot exclude the scenario in which the repertoires of SMs in the preculture- and spore-initiated cocultures are not equivalent. Surprisingly, this bioprocess-related aspect of cocultures still requires systematic and rigorous characterization. Firstly, the effects of switching from precultures to spore suspensions (or vice versa) ought to be investigated with regard to the broad spectrum of products, not merely the industrially relevant molecules, as the levels of unwanted by-products are of great importance during the isolation and purification of target compounds. Secondly, the differences between the spore- and preculture-initiated cocultures should be described both for the simultaneous and the time-separated inoculation, i.e., for the coculture initiation strategies that are frequently used and described in literature. The latter approach is usually applied to prevent the faster growing species from overgrowing its microbial partner. Finally, there is a need to compare the axenic cultures (monocultures) and cocultures of filamentous fungi and actinomycetes regarding the effects resulting from the change of inoculum type. The intention behind the present work was to address these issues by investigating the model coculture of two well-studied filamentous producers of industrially relevant SMs, namely *Aspergillus terreus* and *Streptomyces rimosus* [20–23]. The former microorganism biosynthesizes lovastatin, a cholesterol lowering drug [24], whereas the latter provides an antibiotic drug known as oxytetracycline [25]. In addition, both species generate a wide spectrum of structurally diverse SMs, which are regarded as the unwanted by-products of lovastatin- or oxytetracycline-oriented bioprocesses [26, 27]. Nevertheless, it cannot be excluded that at least some of them will prove useful in future due to their bioactivity.

The aim of this short study was to determine how changing the inoculum type from preculture to spore suspension affects the production of secondary metabolites in submerged cocultures of *A. terreus* and *S. rimosus*.

## Materials and Methods

### Microorganisms

*Aspergillus terreus* ATCC 20542 and *Streptomyces rimosus* ATCC 10970 were used in the study.

### Cultivation on Agar Slants

The 10-day cultivation on agar media (in the form of agar slants) was employed to obtain the spores required to inoculate the liquid medium. In the case of *A. terreus*, the following composition was used for agar slants: malt extract (20 g L^−1^); casein peptone (5 g L^−1^); and agar (20 g L^−1^). For *S. rimosus*, the ISP2 medium (BD, USA) was purchased and prepared according to the manufacturer’s instructions. The temperature in the incubator was maintained at 26 °C.

### Cultivation in Liquid Medium

The composition of the liquid medium was as follows: glucose, 20 g L^−1^; lactose, 20 g L^−1^; yeast extract, 5 g L^−1^; biotin, 0.04 mg L^−1^; NaCl, 0.4 g L^−1^; MgSO_4_·7 H_2_O, 0.5 g L^−1^; KH_2_PO_4_, 1.5 g L^−1^; ZnSO_4_·7 H_2_O, 1 mg L^−1^; Fe(NO)_3_·9 H_2_O, 2 mg L^−1^; and solution of trace elements, 1 mL L^−1^. Composition of trace elements solution: Na_2_MoO_4_·2 H_2_O, 50 mg L^−1^; H_3_BO_3_, 65 mg L^−1^; CuSO_4_·5 H_2_O, 250 mg L^−1^; and MnSO_4_·7 H_2_O, 43 mg L^−1^. The pH was adjusted to 6.5 using 1-M NaOH solution.

The liquid medium cultures were propagated in 500-mL flat-bottom glass flasks. The Innova S44i (Eppendorf, Germany) laboratory shaker was employed throughout the study.

The spores of *A. terreus* and *S. rimosus* were transferred to liquid medium using a sterile 1-mL pipette to achieve (1.0 ± 0.1) · 10^9^ spores L^−1^. The concentration of spores was monitored using a hemocytometer. In the case of 1-stage cocultures and monocultures (i.e., the ones that were inoculated with spore suspensions), 10 mL of each strain’s spore suspension was added to 200 mL of sterile liquid medium, and the inoculated medium was shaken for 168 h (120 rpm, 26 °C). In the case of 2-stage cultures (i.e., the ones that were inoculated with precultures), the inoculated liquid medium was shaken for 24 h and then served as a preculture for the inoculation of a fresh portion of sterile liquid medium. This procedure involved the transfer of 10 mL of preculture of each microorganism into 200 mL of sterile medium. After the completion of the inoculation procedure, the 2-stage cultures were shaken for 168 h (120 rpm; 26 °C). In the monocultures, only one microorganism was inoculated.

### Analysis of SM Production

The samples of fermentation broth were filtered to remove the biomass and the filtrates were subjected to UPLC-MS analysis. The AQUITY-UPLC system (WATERS, USA) with BEH Shield RP18 (2.1 mm × 100 mm × 1.7 μm) column was employed for the chromatographic separation of SMs. The flow rate of eluents through the column was set to 0.2 mL min^−1^. The temperature in the sample manager unit was maintained at 12 °C, while the column temperature was kept at 40 °C. The following H_2_O:acetonitrile gradient was applied (both eluents were acidified with formic acid at 0.1% vol vol^−1^): 0 min, 100:0; 2.5 min, 80:20; 5.5 min, 70:30; 7.5 min, 60:40; and 14.0 min, 40:60. The identification of SMs was performed with the use of SYNAPT G2 high-resolution mass spectrometer (WATERS, USA). The electrospray ionization under negative mode (ESI^−^) was applied (temperature of desolvation, 200 °C; temperature of the source, 120 °C; capillary voltage, 3 kV; sampling cone, 40 V; extraction cone, 4 V).

The identities of mevinolinic acid (lovastatin acid), (+)-geodin, butyrolactone I, and oxytetracycline were confirmed using the reference standards. The remaining SMs were putatively annotated with the use of The Natural Product Atlas [28] by considering the difference between the experimental *m/z* and calculated *m/z* values (the difference never exceeded 0.01) and the previously reported assignment of these molecules to the SM repertoires of *A. terreus* or *S. rimosus* (see [20, 22] and references therein). The standards of mevinolinic acid, (+)-geodin, and oxytetracycline were obtained from Sigma-Aldrich (USA), and butyrolactone I was purchased from Enzo Life Sciences (USA). The UPLC-MS analysis, including peak area determination, was carried out by employing the TargetLynx software (WATERS, USA).

The following products, previously reported by our group in the context of the *A. terreus* vs. *S. rimosus* cocultures [20, 22, 23], were identified or putatively identified over the course of analysis (the presented *m/z* values correspond to [M–H]^−^ ions): oxytetracycline (calculated *m/z*: 459.1404, found *m/z*: 459.1427), mevinolinic acid (calculated *m/z*: 421.2590, found *m/z*: 421.2598), (+)-geodin (calculated *m/z*: 396.9882, found *m/z*: 396.9890), butyrolactone I (calculated *m/z*: 423.1444, found *m/z*: 423.1462), putative 2-decarboxamido-oxytetracycline (ADOTC) (calculated *m/z*: 458.1451, found *m/z*: 458.1409), putative rimocidin (calculated *m/z*: 766.4014, found *m/z*: 766.3990), putative CE-108 (calculated *m/z*: 738.3701, found *m/z*: 738.3635), putative rimocidin (27-ethyl) (calculated *m/z*: 752.3857, found *m/z*: 752.3879), putative milbemycin β_11_ + 4[O] (calculated *m/z*: 593.2962, found *m/z*: 593.3038), putative metabolite resulting from the elimination of “CH_2_O_2_” from rimocidin, i.e., oxidized rimocidin (calculated *m/z*: 720.3959, found *m/z*: 720.3937), putative 4a,5-dihydromevinolinic acid (calculated *m/z*: 423.2747, found *m/z*: 423.2722), putative 3α-hydroxy-3,5-dihydromonacolin L (calculated *m/z*: 339.2172, found *m/z*: 339.2166), putative (+)-erdin (calculated *m/z*: 382.9725, found *m/z*: 382.9689), and putative 4',8''-diacetoxy butyrolactone VI (calculated *m/z*: 523.1610, found *m/z*: 523.1614). In addition, the *m/z* values corresponding to the unknown molecules were detected, namely *m/z* = 457.1589, 599.1751, and the previously reported value of 581.1630 [22].

### Microscopic Observations

All microscopic observations were carried out with the use of OLYMPUS BX53 microscope, OLYMPUS DP27 high-resolution RGB camera, and the software OLYMPUS cellSens Dimension Desktop 1.16 (Olympus Corporation, Tokyo). The projected area (A) values of pellets were determined based on the microscopic images of multiple objects, according to the methods described in the previous work focused on the morphological development in cocultures [21].

### Determination of Biomass Levels

To determine the biomass concentration, 40 mL of the culture was applied onto a pre-weighed Munktell filter disk (grade 389, diameter 150 mm, 84 g m^−2^). After filtration performed under atmospheric pressure, the biomass was washed with 400 mL of distilled water. Then, the filter disk with washed biomass was dried at 105 °C for 24 h and weighed.

### Statistical Analysis

The experiments were carried out in three independent runs. For each experimental variant, the values of product concentration, product peak area, biomass concentration and projected area (A) of pellets were reported as mean ± standard deviation. The statistical significance of differences between the reported values was evaluated by performing the two-sample *t* tests (significance level α = 0.05). All calculations were carried out with the use of OriginPro software (OriginLab Corporation, USA).

## Results

**Fig. 1:**
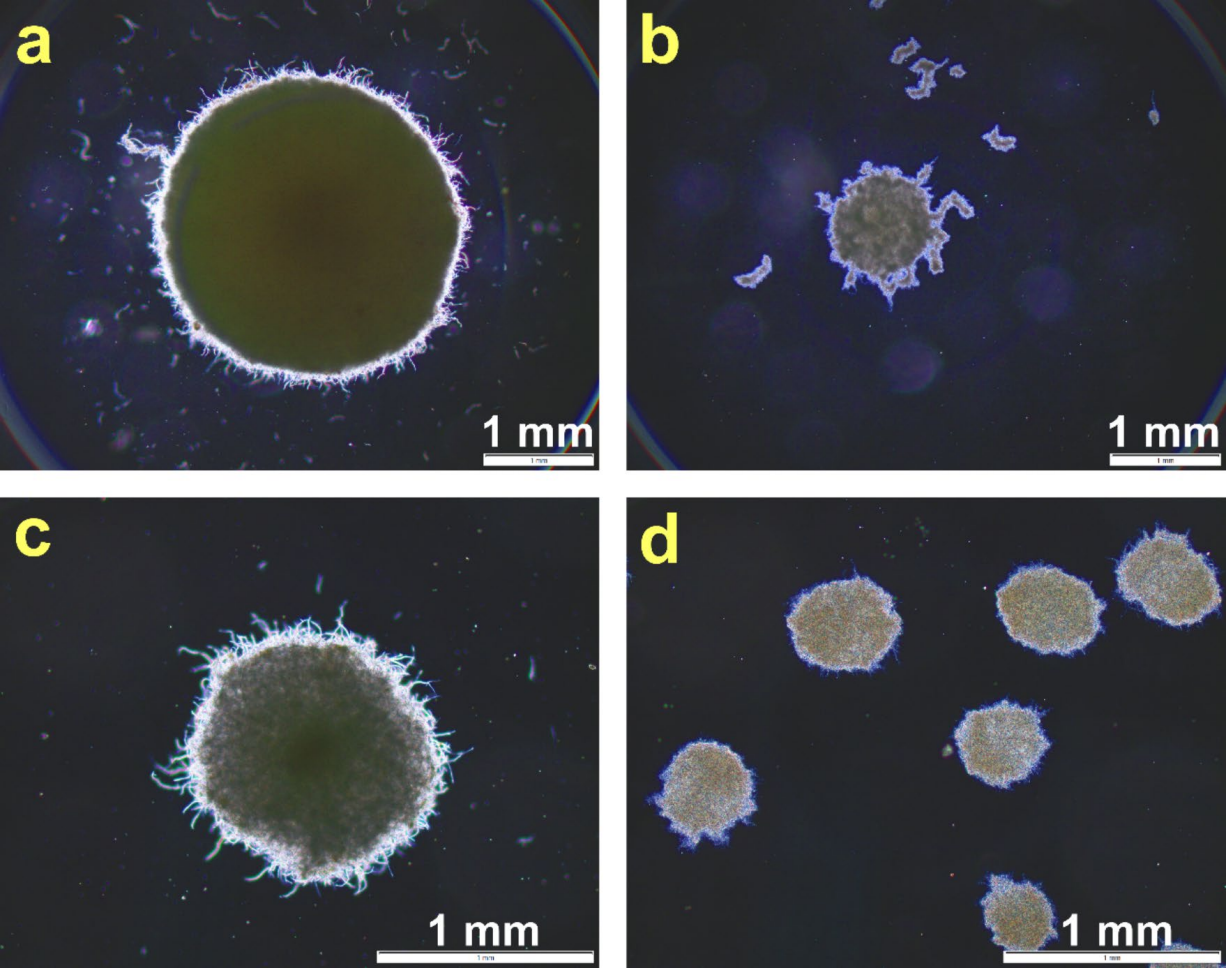
Examples of microscopic images of filamentous pellets observed in the monocultures initiated using the precultures of *A. terreus* (**a**) and *S. rimosus* (**b**) and the monocultures inoculated with spore suspensions of *A. terreus* (**c**) and *S. rimosus* (**d**). The images were recorded 168 h after the inoculation process

In the case of the preculture-based (i.e., 2-stage) cultivations, the filamentous biomass pellets were transferred from the 24-h preculture (i.e., from partially depleted medium) to a portion of fresh medium, as opposed to the spore-based (i.e., 1-stage) processes. It was clearly visible (even during the preliminary macroscopic examination) that the pellets of *A. terreus* and *S. rimosus* formed in the preculture-based monocultures (Fig. 1a, b) were larger than the ones recorded in their spore-based counterparts (Fig. 1c, d). Specifically, the projected area values of *A. terreus* were equal to *A* = (7.3 ± 0.6) · 10^6^ μm^2^ and *A* = (1.2 ± 0.3) · 10^6^ μm^2^ for the preculture- and spore-initiated monocultures, respectively (*P* < 0.0001). In the case of *S. rimosus* pellets, the values recorded for the monocultures initiated with the use of preculture or spore suspension were *A* = (1.0 ± 0.1) · 10^6^ μm^2^ and *A* = (1.4 ± 0.4) · 10^5^ μm^2^, respectively (*P* < 0.0001).

The growth and morphology of *A. terreus* and *S. rimosus* in cocultures were assessed by performing microscopic observations (Fig. S1) and determining the biomass concentration (Fig. S2). The biomass growth was confirmed in all tested variants, with the highest mean biomass level (5.9 g L^−1^) reached in the *A. terreus* monoculture initiated with the use of preculture (Fig. S2). In the case of pellets formed in spore-initiated cocultures, the morphology in the simultaneously inoculated coculture (Fig. S1a) resembled the one recorded in the *S. rimosus* monoculture (Fig. 1d), especially regarding the projected area of the pellets (*P* > 0.05). To be specific, the projected area for the spore-based coculture was equal to *A* = (1.2 ± 0.9) · 10^5^ μm^2^. However, when the precultures were used for coculture initiation, one could observe that both the *A. terreus*-like and the *S. rimosus*-like pellets were present in the simultaneously inoculated cocultures (Fig. S1b). When the delayed inoculation was applied, only the morphological structures resembling the first-inoculated microorganism were visible, while the delayed strain was practically undetectable (Fig. S1c–j). This observation remained valid regardless of the inoculum type (i.e., spores or precultures) and the time of delay (i.e., 24 or 48 h). For example, in the preculture-initiated cocultures, the 24-h delay of *S. rimosus* inoculation relative to *A. terreus* introduction resulted in the formation of *A. terreus*-like pellets (Fig. S1g) that were visibly larger, with *A* = (6.9 ± 0.7) · 10^6^ μm^2^, *P* < 0.0001, than the pellets recorded for the corresponding *S. rimosus* monocultures (Fig. 1b). The pellets were not the only morphological form detected in the broth. They were accompanied by loose filaments and clumps; however, the origin of these structures was not investigated here as the morphologies were not monitored on a daily basis but only upon bioprocess completion. So, it was unknown whether their presence was due to pellet “shaving” over the course of cultivation or if the mixed morphology was already established in the initial phase of the process.

**Fig. 2:**
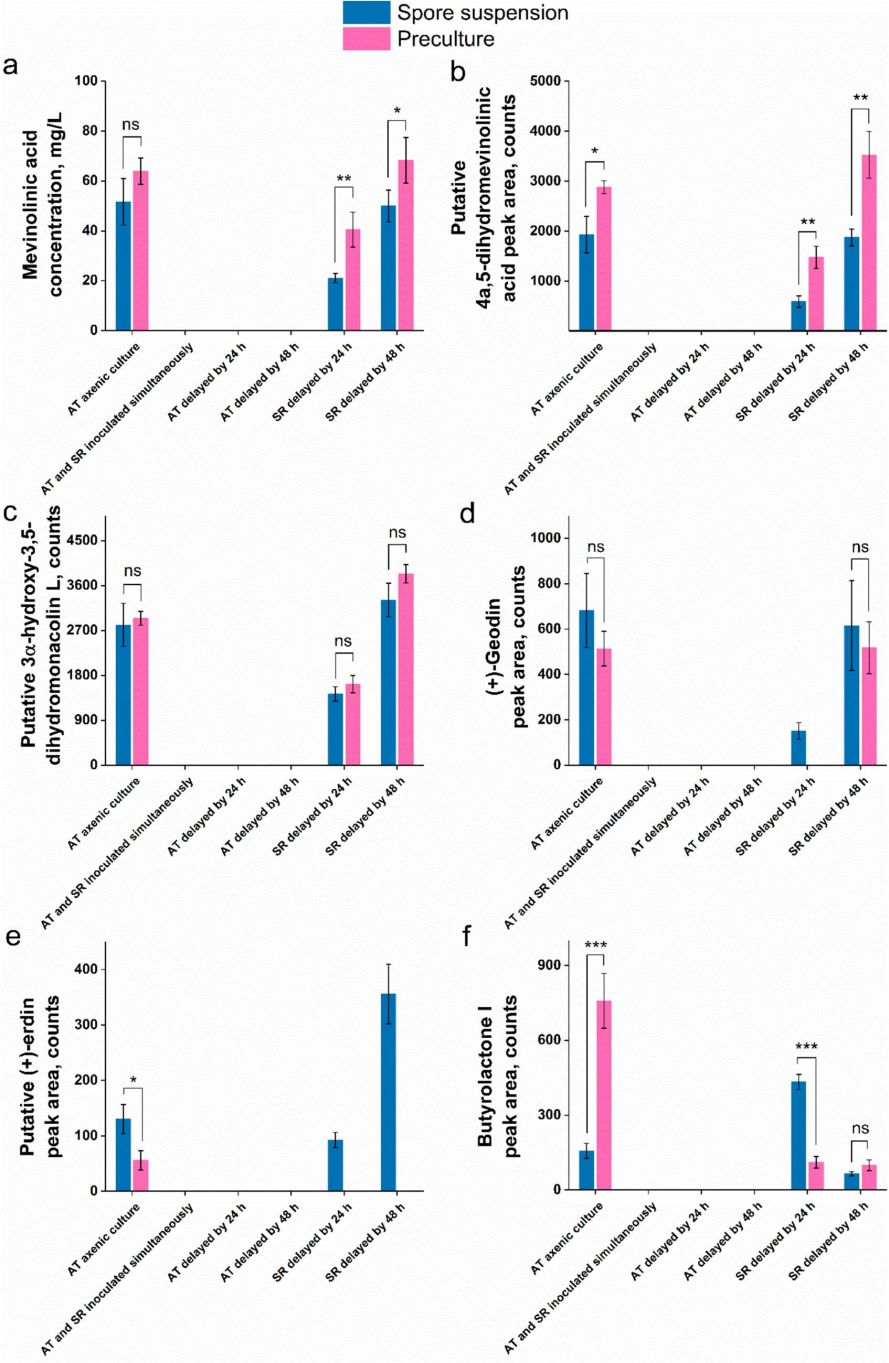
The levels of secondary metabolites produced by *A. terreus* in the axenic cultures (monocultures) and cocultures involving *S. rimosus* and *A. terreus*, namely mevinolinic acid (**a**), putative 4a,5-dihydromevinolinic acid (**b**), putative 3α-hydroxy-3,5-dihydromonacolin L (**c**), (+)-geodin (**d**), putative (+)-erdin (**e**), and butyrolactone I (**f**). The identities of mevinolinic acid (**a**), (+)-geodin (**d**), and butyrolactone I (**f**) were confirmed with the use of authentic standards, while the remaining metabolites were putatively annotated by considering the agreement of *m/z* values (the difference with respect to the calculated *m/z* value was always less than 0.01) and previous literature reports on *A. terreus* biosynthetic repertoire. The detected products were previously reported in the context of cocultures involving *A. terreus* and *S. rimosus* [20, 22, 23]. Each result represents the mean ± standard deviation (data from three experiments). The statistical significance of differences between the preculture- and spore-initiated cultures was determined using the two-sample *t* test. *, *P* < 0.05; **, *P* < 0.01; ***, *P* < 0.001; *ns* not significant, *AT* *A. terreus*, *SR* *S. rimosus*

Altogether, six SMs biosynthesized by *A. terreus* were detected in the broth, including three products with confirmed identities, namely mevinolinic acid (i.e., lovastatin in its acidic form), (+)-geodin, and butyrolactone I. Each of these SMs represents a distinct biosynthetic pathway previously characterized in *A. terreus*. Most importantly, the effect of inoculum type on the SM production was observed to be pathway-specific. In the case of mevinolinic acid (Fig. 2a), its derivative 4a,5-dihydromevinolinic acid (Fig. 2b), and the precursor molecule known as 3α-hydroxy-3,5-dihydromonacolin L (Fig. 2c), the mean levels obtained in the preculture-initiated cultures were higher than in the corresponding variants inoculated with spores (both for the mono- and cocultures), yet the differences were not always found to be statistically significant. The opposite observations were made with regard to (+)-geodin (Fig. 2d) and its derivative (+)-erdin (Fig. 2e), as these two octaketide metabolites reached relatively higher mean levels in the spore-initiated cultures, regardless of whether the mono- or cocultures were considered. By contrast, the production of butyrolactone I (Fig. 2f) did not follow the correlations displayed by the SMs generated via the mevinolinic acid or (+)-geodin pathways. In the monocultures, the preculture-based fermentation led to markedly higher butyrolactone I levels than its spore-initiated counterpart. In the case of the cocultures, the 24-h delay of *S. rimosus* relative to *A. terreus* resulted in the spore-based cocultivation yielding higher product levels than the process initiated with the use of the preculture. When the actinomycete delay was equal to 48 h, there were no significant differences between the spore- and preculture-based cocultures (Fig. 2f). The common behavior recorded for all the detected SMs of *A. terreus* was that they were absent in the cocultures initiated through simultaneous inoculation of *A. terreus* and *S. rimosus*. In addition, they were undetectable in the cocultures for which the inoculation of *A. terreus* was delayed for 24 or 48 h (Fig. 2).

**Fig. 3:**
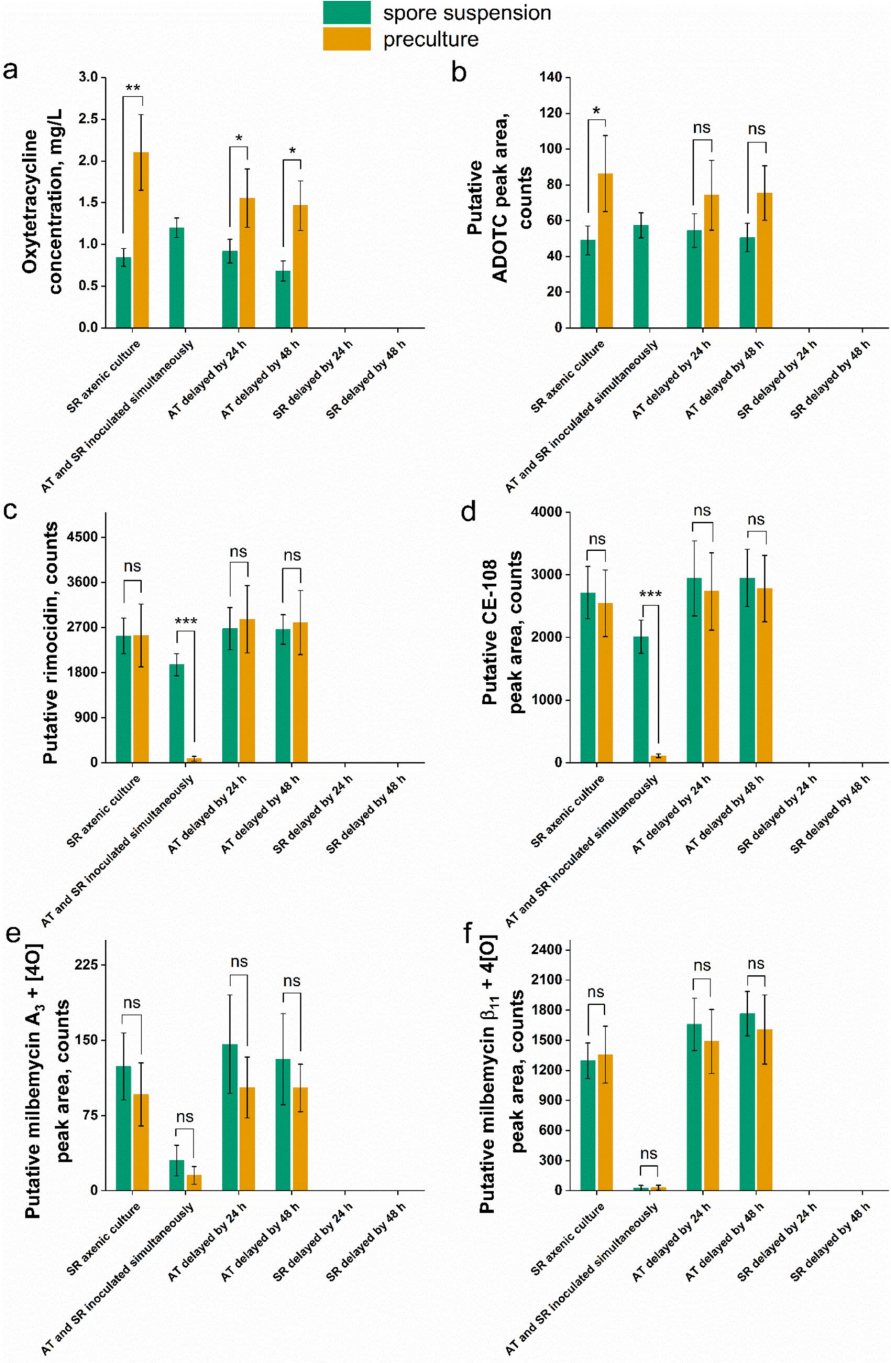
The levels of secondary metabolites produced by *S. rimosus* in the axenic cultures (monocultures) and cocultures of *S. rimosus* and *A. terreus*, namely oxytetracycline (**a**), putative 2-decarboxamido-oxytetracycline (ADOTC) (**b**), putative rimocidin (**c**), putative CE-108 (**d**), putative milbemycin A_3_ + 4[O] (**e**), and putative milbemycin β_11_ + 4[O] (**f**). The identity of oxytetracycline (**a**) was confirmed with the use of authentic standard, while the remaining metabolites were putatively annotated by considering the agreement of *m/z* values (the difference with respect to the calculated *m/z* values was always less than 0.01) and previous literature reports on *S. rimosus* biosynthetic repertoire. The detected products were previously reported in the context of cocultures involving *A. terreus* and *S. rimosus* [20, 22, 23]. Each result represents the mean ± standard deviation (data from three experiments). The statistical significance of differences between the preculture- and spore-initiated cultures was determined using the two-sample *t* test. *, *P* < 0.05; **, *P* < 0.01; ***, *P* < 0.001; *ns* not significant, *AT* *A. terreus*, *SR* *S. rimosus*

Regarding the metabolic products of *S. rimosus*, six SMs were found in the fermentation broth over the course of the experiment, including oxytetracycline and five putatively identified molecules representing the rimocidin and milbemycin families (Fig. 3). Importantly, their biosynthesis was also confirmed in the majority of the cocultures initiated via the simultaneous inoculation of *A. terreus* and *S. rimosus*, what contrasted with the observations on *A. terreus* metabolites, as they were always absent in these coculture variants (Fig. 2). If the cocultures were initiated by the simultaneous introduction of *A. terreus* and *S. rimosus* spores into the medium, all six SMs of *S. rimosus* were detected. However, if the preculture was used instead of spores, the simultaneously inoculated cocultures yielded no oxytetracycline (Fig. 3a) and no ADOTC (Fig. 3b), while the levels of rimocidin (Fig. 3c) and CE-108 (Fig. 3d) were strikingly lower than in the spore-based coculture counterparts. In the case of the SMs putatively identified as milbemycins, both the spore- and preculture-based cocultures initiated via the simultaneous inoculation yielded relatively low product levels (Fig. 3e, f) and the preculture vs. spores comparison did not reveal any statistically significant differences. As far as the *S. rimosus* monocultures and the cocultures involving the delayed inoculation of *A. terreus* were concerned, replacing the preculture inoculum with spore suspension rarely resulted in the significant change in SMs levels. More specifically, the differences between the preculture- and spore-initiated cocultures were negligible in the case of rimocidins (Fig. 3c, d) and milbemycins (Fig. 3e, f), however, the production of oxytetracycline, the pharmaceutically relevant SM of *S. rimosus*, clearly benefited from the inoculation with precultures in the *A. terreus*-delayed variants (Fig. 3a). Hence, these results contrasted strongly with the data obtained for the simultaneously inoculated coculture variants, which showed the non-zero levels of oxytetracycline (Fig. 3a) and ADOTC (Fig. 3b) only if the spores were used for coculture initiation.

**Fig. 4:**
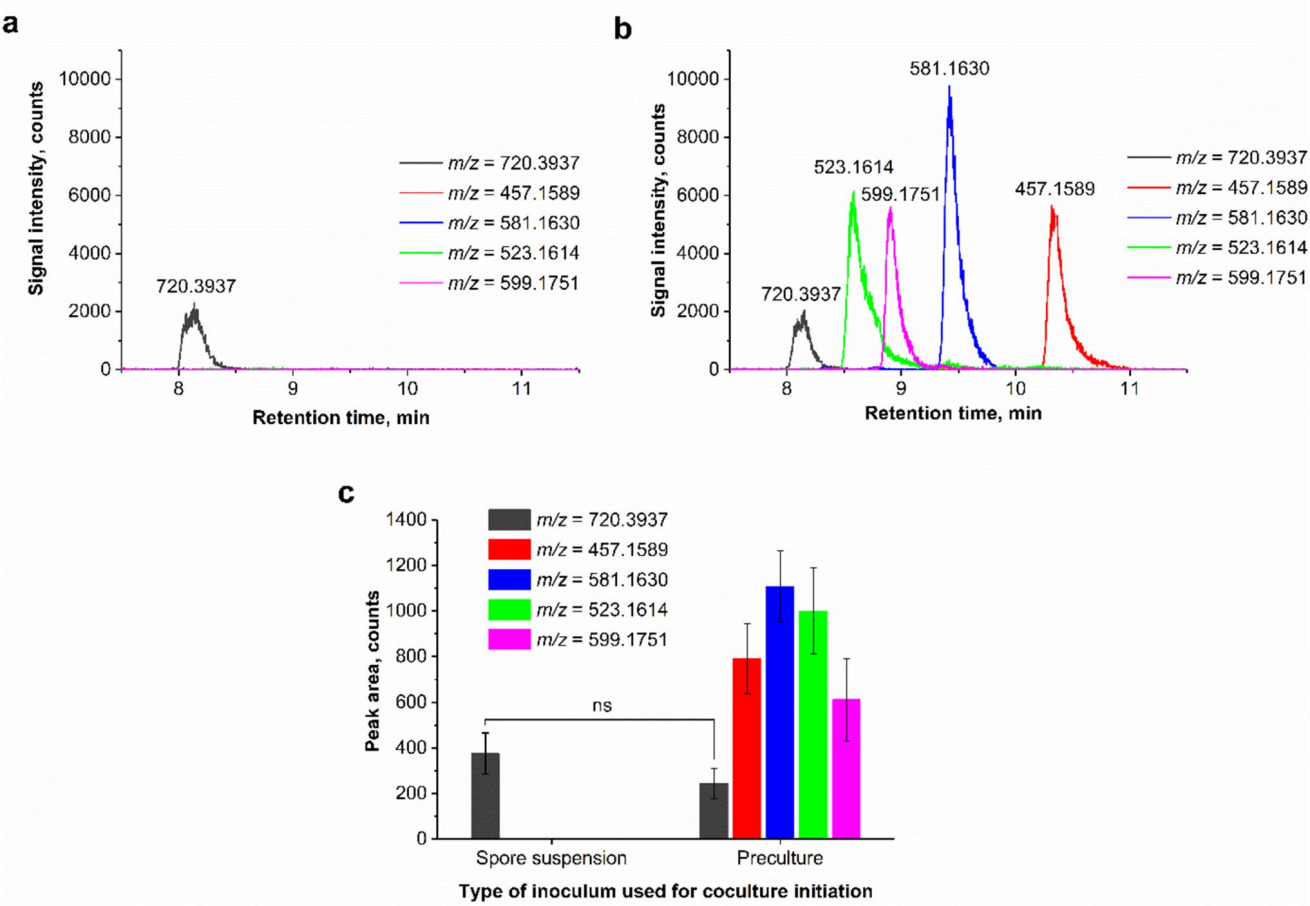
The results regarding the secondary metabolites detected solely in the cocultures initiated through the simultaneous inoculation of *A. terreus* and *S. rimosus* (no traces of these molecules were found in the monocultures and the cocultures involving the delayed inoculation of one of the strains). The extracted ion chromatograms (under ESI^−^ mode) of products displaying the *m/z* values of 720.3937, 457.1589, 581.1630, 523.1614, and 599.1751 were generated for the cocultures initiated with the use of spore suspensions (**a**) and precultures (**b**). The peak area values corresponding to these *m/z* values were also determined (**c**) to compare the product levels reached in the spore- and preculture-based cocultures. The products characterized by the *m/z* values of 720.3937 and 523.1614 were previously reported for the *A. terreus* and *S. rimosus* cocultures and putatively annotated as the molecule resulting from the elimination of “CH_2_O_2_” group from rimocidin (i.e., oxidized rimocidin), and 4′,8″-diacetoxy butyrolactone VI, respectively [20, 22, 23]. The *m/z* value of 581.1630, corresponding to an unknown metabolite, was previously recorded by Boruta et al. [22]

The ESI^−^ analysis revealed the presence of five SMs which were detected in the cocultures initiated via the simultaneous inoculation of *A. terreus* and *S. rimosus* but were absent in all the remaining experimental variants, i.e., in the monocultures and the cocultures involving the delayed inoculation of the fungus or the actinomycete. Upon further investigation it became clear that among these five coculture-specific products only one (with *m/z* = 720.3937) was confirmed both in the spore- and preculture-based cocultures. The remaining four molecules (*m/z* = 457.1589, 581.1630, 523.1614, and 599.1751) were found exclusively in the cocultures initiated with preculture (Fig. 4). This observation was confirmed by analyzing the extracted ion chromatograms (Fig. 4a, b) and peak area values (Fig. 4c). The products of *m/z* = 720.3937 and 523.1614 were discovered previously as the coculture-specific products and putatively annotated, respectively, as the molecule resulting from the elimination of “CH_2_O_2_” group from rimocidin, i.e., oxidized rimocidin [20, 22, 23], and the member of the butyrolactone family, 4′,8″-diacetoxy butyrolactone VI [22]. In addition, a previously reported *m/z* value of 581.1630, corresponding to the molecule of unknown identity found in *A. terreus* and *S. rimosus* coculture [22] was detected here. To the best of our knowledge, the remaining two SMs (i.e., the ones with *m/z* = 457.1589 and 599.1751) have not been yet reported in the context of *A. terreus* or *S. rimosus* biosynthetic repertoires.

## Discussion

According to the results of the study, the repertoires and quantities of SMs in the *A. terreus* vs. *S. rimosus* cocultures clearly depended on the inoculum type. If the study had been performed solely with the use of spore suspensions, 4 out of 5 coculture-specific SMs (Fig. 4) would not have been discovered, as they appeared exclusively in the preculture-based cocultures. On the other hand, the presence of (+)-erdin in the inoculation-delayed cocultures (Fig. 2e), and oxytetracycline in the simultaneously inoculated cocultures (Fig. 3a) was revealed only in the case of spore-initiated cocultures. Based on these observations, the diversification of inoculum types used for coculture initiation is suggested here to effectively uncover the full biosynthetic potential of *Aspergillus*, *Streptomyces,* and other filamentous genera.

The typical issue discussed in the SM-oriented coculture studies is the comparison between the cocultures and their monoculture counterparts in terms of target product titers. The present study proved that inoculum type is among the factors that determine the outcomes of microbial cocultivation processes. In other words, the conclusions drawn on the basis of preculture-based cocultures may differ considerably from the observations made with regard to the spore-based coculture bioprocesses. For example, the production of butyrolactone I in the cocultures was always less effective than in the *A. terreus* monocultures provided the preculture was used for process initiation (Fig. 2f). This observation was no longer valid if the preculture was replaced with spores. Another question often formulated over the course of microbial coculture studies is whether the coculture approach shows any potential in the context of stimulating SM production (i.e., increasing their levels) compared with the monoculture. Again, following the experimental protocol involving only a single inoculum type may in some cases be misleading. The biosynthesis of (+)-erdin serves as an example to illustrate this point. The use of preculture for the inoculation ultimately revealed no chances of achieving the cocultivation-related boost of (+)-erdin production, whereas employing the spore-based inoculum resulted in much more promising outcomes, i.e., the elevated levels of (+)-erdin in the *S. rimosus*-delayed cocultures relative to the *A. terreus* monoculture (Fig. 2e). Hence, the conclusions regarding the effect of cocultivation on (+)-erdin biosynthesis were visibly dependent upon the inoculum type.

For the cocultures initiated though the simultaneous inoculation of *A. terreus* and *S. rimosus*, the type of inoculum had a profound effect on the SM production-related outcomes. Firstly, the biosynthesis of oxytetracycline (Fig. 3a) and its derivative ADOTC (Fig. 3b) was confirmed only in the spore-initiated cocultures, while being absent in the preculture-based variants. Secondly, the formation of rimocidin (Fig. 3c) and CE-108 (Fig. 3d) was markedly stimulated in the spore-based coculture compared with its preculture-based counterpart. Finally, the study revealed four SMs that were produced only in the coculture initiated via the simultaneous introduction of precultures (not spores) into sterile medium (Fig. 4). Apparently, the developed filamentous biomass structures of both species were required to yield the new SMs, as opposed to the product represented by the *m/z* value of 720.3937, which appeared regardless of whether the preculture or spore inoculum was used. In the latter case, the spores of the slower-growing species (i.e., *A. terreus*) were likely to be “engulfed” inside the developing pellets of faster-growing microorganism (i.e., *S. rimosus*) [29]. Still, the presence of the coculture-specific metabolite implies that the fungus, despite being outperformed by the actinomycete, played a role in expanding the SM repertoire of the coculture. It was suggested previously that the product represented by the *m/z* value of 720.3937 could be an effect of fungal biotransformation of rimocidin [20].

In the simultaneously inoculated cocultures, the majority of detected *S. rimosus* metabolites reached higher levels in the spore-based variants (Fig. 3), while the use of precultures led to the shutdown or inhibition of their production. At the same time, the formation of *A. terreus* SMs in the simultaneously inoculated cocultures was non-existent (Fig. 2). Hence, the pellets of *A. terreus* and *S. rimosus* displayed mutual inhibition in terms of their biosynthetic capabilities. At the same time, the simultaneous inoculation with spores resulted in the dominance of *S. rimosus* over the fungus, as manifested by the lack of *A. terreus* SMs (Fig. 2) and the presence of *S. rimosus* SMs (Fig. 3). All in all, the pellets of *A. terreus* did show the ability to inhibit the pellets of *S. rimosus*, yet in the spore-initiated cocultures the dominance of *S. rimosus* over *A. terreus* was evident, provided the spores of both species were introduced simultaneously.

When *A. terreus*, either in the form of spore suspension or preculture, was introduced with a 24-h or 48-h delay relative to *S. rimosus*, the SMs of *A. terreus* were not detected in the broth (Fig. 2). Analogously, no products of *S. rimosus* could be found in the cocultures for which the actinomycete inoculation took place 24 or 48 h after the introduction of the fungus (Fig. 3). The time advantage given to one of the strains resulted in the scenario in which the delayed strain was outcompeted, and its SM production was not manifested. The already established culture of the first-inoculated microorganism constituted an inhibitory environment for the second strain and replacing the spore suspension with the filamentous pellet-containing preculture did not reverse this effect.

In the previously reported studies, the use of spore suspension inoculum led to higher concentrations of actinorhodin and natamycin in the monocultures of *Streptomyces coelicolor* [19] and *Streptomyces natalensis* [18], respectively, compared with the cultivations initiated with the precultures. Here, as far as the monocultures of *S. rimosus* were concerned, the production of oxytetracycline (Fig. 3a) and ADOTC (Fig. 3b) turned out to be enhanced in the preculture-based variants, while the levels of putative rimocidins (Fig. 3c, d) and milbemycins (Fig. 3e, f) were not visibly influenced by the inoculum type. Hence, there are no theory-based recommendations regarding the choice of inoculum type that may be universally applied to all SM-aimed filamentous microbial monocultures. Importantly, this remark remains valid also with respect to the cocultures.

Regarding the SM production in *A. terreus* vs. *S. rimosus* cocultures, Boruta et al. [20] previously described nine variants of cocultures propagated in stirred tank bioreactors. Only one coculture was initiated with the use of spores, while in the remaining eight variants the precultures were employed. The bioreactor-based study was focused on the SM repertoire and process-related differences between various monocultures and cocultures, it was not designed to rigorously investigate the spore suspension vs. preculture issue. Importantly, the dissolved oxygen level was controlled at 20% of saturation via the automatic adjustment of stirring speed and air flow rate, hence the hydrodynamic conditions were not equivalent throughout the investigated bioreactor runs, what in turn exerted the effect on SM production [20] and, as later analyzed by Ścigaczewska et al. [21], on the morphological characteristics. Furthermore, the spore inoculum was used solely in the variant initiated through the simultaneous inoculation, the 48-h delays were not performed at all, and the delay of *A. terreus* inoculation relative to *S. rimosus* was not attempted [20]. In the present study, the experiment was designed to compare the effects of different inocula, so the cultivations were all performed at the constant speed of the rotary shaker in shake flasks. What is more, two variants of inoculation time delay were considered here for comparative purposes, i.e., the 24-h and 48-h delays, and the delays of *A. terreus* and *S. rimosus* were both investigated and compared. Even though the bioprocess conditions in the previously described experiments and the present work were not equivalent, it is important to address the main similarities and differences with regard the recorded results. Notably, in the cocultures inoculated simultaneously with the use of precultures the *A. terreus*-like and *S. rimosus*-like pellets were both observed regardless of whether the bioreactor [21] or flasks (as in the present work) were employed for cultivation. However, the production of SMs by *S. rimosus* in the bioreactor coculture was comparable with the production exhibited by the corresponding *S. rimosus* bioreactor monoculture [20], whereas in the present study, the inhibition of *S. rimosus* biosynthetic activity was observed in the coculture. As already mentioned in the previous work [22], the outcome of cocultivation is greatly dependent on the process-related factors (agitation, aeration, shear stress, scale, etc.) that collectively affect the biosynthesis of SMs.

The biosynthesis of microbial SMs is governed by complex regulatory mechanisms that are still far from being fully understood. The multi-level regulation of coelimycin production in *Streptomyces coelicolor* A3(2) illustrates the level of complexity typically associated with SM formation [30]. It is known that the regulatory networks in *Streptomyces* involve a broad range of global and pathway-specific regulators, respond to signals associated with carbon, nitrogen and phosphate sources, and are linked to the morphological development of the microorganism. In addition, the γ-butyrolactone (GBL) regulatory systems were shown to be involved in the biosynthesis of antibiotics in *Streptomyces* [31]. For example, the so-called A-factor (2-isocapryloyl-3*R*-hydroxymethyl-γ-butyrolactone) triggers cell differentiation and streptomycin biosynthesis in *Streptomyces griseus* [32]. As far the SM production in *A. terreus* is concerned, the regulatory mechanisms involved in SM production in filamentous fungi also display great complexity and involve multi-level control of biosynthetic processes [1, 33]. In the context of the present study, it is important to note that the interactions between microorganisms in coculture may provide the environmental cues that trigger, stimulate or inhibit the production of SMs by affecting the cellular regulatory cascades [34].

In coculture, the strains may respond to the presence of competitors by recognizing the signals associated with the depletion of substrates or the production of molecules that negatively affect or damage the cell (e.g., antibiotics). In addition, the quorum-dependent regulators, such as GBLs, may serve as the indicators of competitor’s presence. These issues have been previously discussed by Westhoff et al. [35] by referring to the production of antibiotics in the genus *Streptomyces*. With regard to quorum sensing, i.e., a cell-to-cell communication mechanism relying on the accumulation of signaling molecules to sense cell density [36], it has been investigated in the context of bacterial and fungal development and metabolism [37, 38]. In the present study, the metabolite previously proposed to act as a quorum sensing molecule in *A. terreus* [39], namely butyrolactone I, was detected in the fermentation broth. Further studies are required to determine if the presence of butyrolactone I, which is referred to as a “γ-butyrolactone-containing” metabolite [40], can be sensed by the cells of *S. rimosus*. To the best of our knowledge, the effect of butyrolactone I on the species representing the genus of *Streptomyces* remains uncharacterized.

In this study, the production of rimocidin, an antifungal metabolite [41] produced by *S. rimosus*, was detected. Furthermore, the presence of (+)-geodin, a well-described octaketide biosynthesized by *A. terreus*, was confirmed. According to the previous report, (+)-geodin shows antibiotic activity against Gram-positive bacteria [42]. While the production of (+)-geodin is not observed during the initial 24 h of cultivation, regardless of whether the mono- or cocultures are analyzed [43, 44], the biosynthesis of rimocidin by *S. rimosus* usually takes place already during the first day of the process [20, 45]. Even though the day-to-day sampling and kinetic characterization were not performed here, one may assume that the fermentation broth already contained SMs at the time of coculture initiation, with the exception of the “*A. terreus* spores vs. *S. rimosus* spores” initiation variant. For example, it was previously noted by our group that the levels of butyrolactone I were relatively high after 48 h of *A. terreus* cultivation, unless the biosynthesis of this metabolite was blocked due to unfavorable process conditions [20]. Our observation agreed with the results of Palonen et al. [46], who reported that the concentration of butyrolactone I in the culture of *A. terreus* “peaked at 48 h post inoculation.”

The differences observed among the tested variants with respect to the exhibited SM repertoire were associated with microbial interactions. As far as the chemical interactions are concerned, the cocultured strains could influence each other through the consumption of substrates and the biosynthesis of SMs. While the influence of SMs on the members of the *S. rimosus* vs. *A. terreus* coculture remains unknown, it is tempting to speculate that each strain was affected by the changes in nutritional environment occurring due to the growth of its microbial partner in coculture. It is important to note that nutrient stress and the composition of growth medium are known to influence the production of microbial SMs [47, 48]. If the coculture is initiated with the use of spores, the observable effect associated with substrate depletion is delayed compared to the preculture-based inoculation scenario, due to the time needed for the germination of spores and the outgrowth of filamentous structures [49]. Using the words of Papagianni and Mattey [50], the spore-based inoculation “shifts fermentation backward” compared to the process initiated with use of preculture. With regard to the spectrum of SMs, it is important to note that their production was previously reported to be dependent on the developmental stage of the microbial producer [51]. Again, reaching detectable biomass and product levels in the spore-based cocultures requires time associated with the development of filamentous morphological forms. Importantly, the SMs produced in the coculture may display antimicrobial activity [52] or act as signaling molecules [53], thus influencing the cocultured strains on the chemical level. The interactions occurring at the physical level are based on the direct contact between the two strains. In the case of the *S. rimosus* vs. *A. terreus* coculture, the process of co-aggregation, previously reported in the context of microbial cocultures [29, 54], might have played the role in shaping the growth- and production-related capabilities of the two strains. Finally, it should be made clear that the effects of different kinds of interspecies interactions on the spectrum of SMs require further research. The present study was focused on describing the outcome of the bioprocess, however, the environmental signals directly responsible for the stimulation or inhibition of SM production in *S. rimosus* vs. *A. terreus* coculture remain to be elucidated.

## Conclusion

Replacing the preculture with spore inoculum positively affects the production of oxytetracyline and the molecules putatively identified as rimocidin and CE-108 in the cocultures initiated through the simultaneous inoculation of *S. rimosus* and *A. terreus*. Compared with the preculture-based variants, the preculture-to-spores replacement also leads to the stimulation of butyrolactone I production and the triggering of (+)-geodin and (+)-erdin biosynthesis in the cocultures for which the introduction of *S. rimosus* is delayed by 24 h relative to *A. terreus*.

The investigated coculture system involving *S. rimosus* and *A. terreus* perfectly demonstrates the need for the diversification of inoculum types in the SM-oriented studies. Employing only precultures or spore suspensions may lead to the underestimation of SM production potential of fungi and actinomycetes, both in qualitative and quantitative terms. Most importantly, depending on the SM, the biosynthesis of a target molecule may be detected solely in relation to one inoculum type or manifest itself regardless of whether the precultures or spores are used for the inoculation. In addition, the conclusions regarding the comparison between the SM production exhibited by the monocultures and the cocultures may differ considerably depending on the type of inoculum. Based on the results of the study, considering different inoculum types is recommended in the comparative studies involving filamentous microbial cocultures and their monoculture counterparts.

## Supplementary Information

Below is the link to the electronic supplementary material.

Supplementary file1 (DOCX 2086 KB)

## Data Availability

The datasets generated during and/or analyzed during the current study are available from the corresponding author on reasonable request.
