# Supplementary material for: Changing the Inoculum Type From Preculture to Spore Suspension Markedly Alters the Production of Secondary Metabolites in Filamentous Microbial Coculture
Source: Curr Microbiol. 2024 Dec 7;82(1):31. doi: 10.1007/s00284-024-04007-x (PMC11625075; doi:10.1007/s00284-024-04007-x)
Supplement: Supplementary file 1 — Supplementary file1 (DOCX 2086 KB) [file 284_2024_4007_MOESM1_ESM.docx]

**Supplementary material**

**Changing the inoculum type from preculture to spore suspension markedly alters the production of secondary metabolites in filamentous microbial coculture**

Tomasz Boruta*, Weronika Pawlikowska, Martyna Foryś, Grzegorz Englart, Anna Ścigaczewska

Lodz University of Technology, Faculty of Process and Environmental Engineering, Department of Bioprocess Engineering, ul. Wólczańska 213, 93-005 Łódź, Poland

* Corresponding author. Phone: +48 42 631 39 77; fax: +48 42 636 56 63

E-mail address: [tomasz.boruta@p.lodz.pl](mailto:tomasz.boruta@p.lodz.pl)


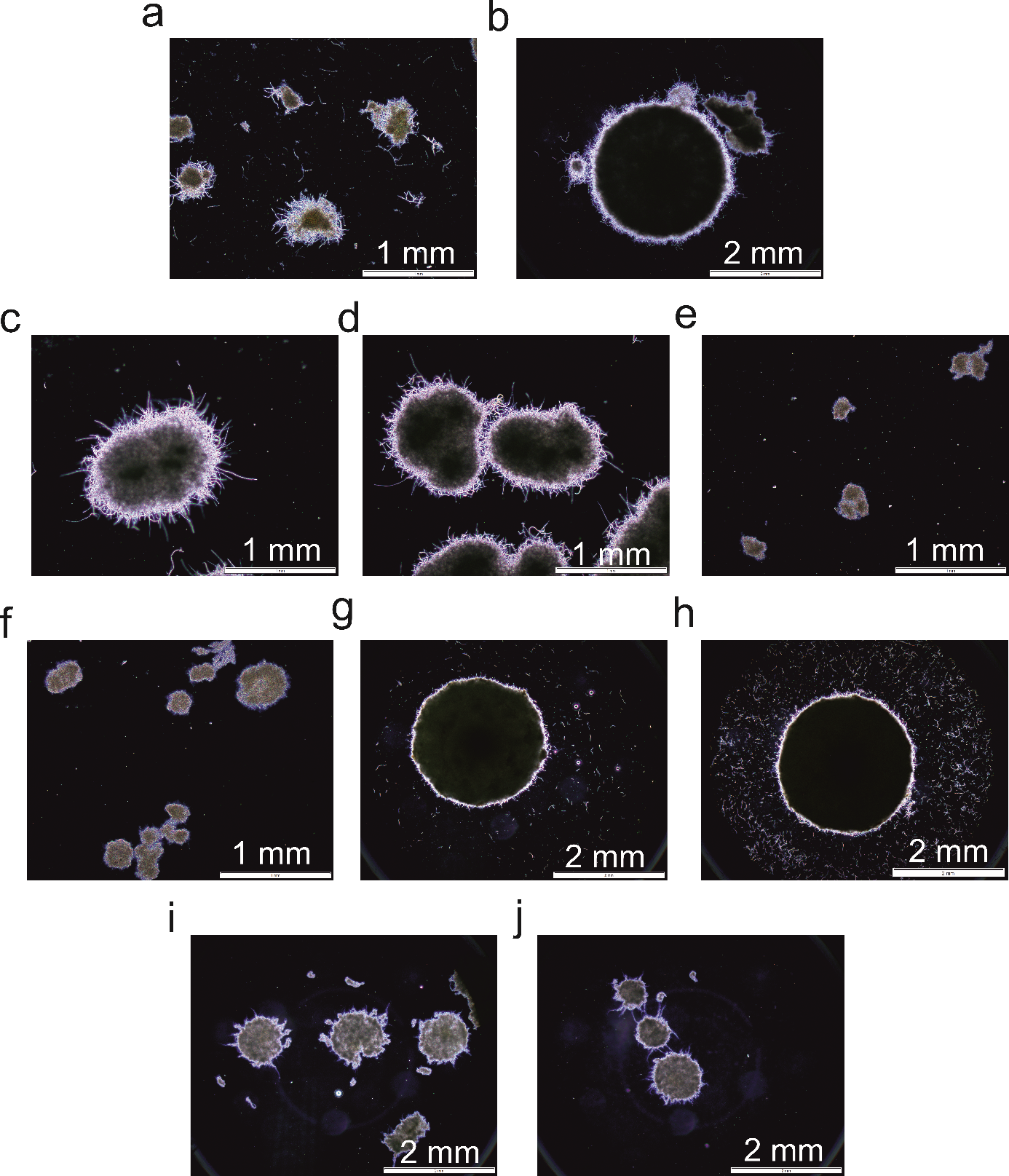


**Fig. S1** Microscopic images depicting the morphological forms in *A. terreus* (AT) and *S. rimosus* (SR) cocultures: (a) AT and SR inoculated simultaneously with the use spores, (b) AT and SR inoculated simultaneously with the use of precultures, (c) SR delayed by 24 h, inoculum: spores, (d) SR delayed by 48 h, inoculum: spores, (e) AT delayed by 24 h, inoculum: spores, (f) AT delayed by 48 h, inoculum: spores, (g) SR delayed by 24 h, inoculum: precultures, (h) SR delayed by 48 h, inoculum: precultures, (i) AT delayed by 24 h, inoculum: precultures, (j) AT delayed by 48 h, inoculum: precultures. The images were recorded 168 h after the inoculation process.





**Fig. S2** The total biomass concentration values recorded for the axenic cultures (monocultures) and cocultures of *A. terreus* (AT) and *S. rimosus* (SR). The results are presented as mean value ± standard deviation (n = 3).
